# Therapeutic efficacy and tolerability of artemether–lumefantrine for uncomplicated *Plasmodium falciparum* malaria in Niger, 2020

**DOI:** 10.1186/s12936-024-04945-8

**Published:** 2024-05-13

**Authors:** Ibrahim M. Laminou, Ibrahima Issa, Eric Adehossi, Kabirou Maman, Hadiza Jackou, Eric Coulibaly, Zilahatou B. Tohon, Jehan Ahmed, Elisha Sanoussi, Daniel Koko

**Affiliations:** 1 Medical and Health Research Centre, Niamey, Niger; 2 Abdou Moumouni School of Health Sciences at University of Niamey-Niger , Niamey, Niger; 3 National Malaria Control Programme, Niamey, Niger; 4 U.S. President’s Malaria Initiative, USAID, Niamey, Niger; 5 U.S. President’s Malaria Initiative Impact Malaria, Atlanta, USA; 6 U.S. President’s Malaria Initiative Impact Malaria, Niamey, Niger

**Keywords:** Resistance, *P. falciparum*, Artemether–lumefantrine, Efficacy, Niger

## Abstract

**Background:**

Monitoring therapeutic efficacy is important to ensure the efficacy of artemisinin-based combination therapy (ACT) for malaria. The current first-line treatment for uncomplicated malaria recommended by the National Malaria Control Program in Niger is artemether–lumefantrine (AL). In 2020, an in vivo study was carried out to evaluate clinical and parasitological responses to AL as well as the molecular resistance to the drug in three sentinel sites: Agadez, Tessaoua and Gaya, in Niger.

**Methods:**

A multi-center, single-arm trial was conducted according to the 28-day World Health Organization (WHO) 2009 therapeutic efficacy study protocol. Children between 6 months and 15 years with confirmed uncomplicated *Plasmodium falciparum* infection and 1000–200,000 asexual parasites/μL of blood were enrolled and followed up for 28 days. Uncorrected and PCR-corrected efficacy results at day 28 were calculated, and molecular correction was performed by genotyping the *msp1, msp2,* and *glurp* genes. The *pfk13, pfdhfr, pfdhps, pfcrt and pfmdr* genes were analyzed by PCR and Sanger sequencing. The Kaplan–Meier curve assessed parasite clearance.

**Results:**

A total of 255 patients were enrolled in the study. The adequate clinical and parasitological response after PCR correction was 98.9% (95% CI 96.4–101.0%), 92.2% (85.0–98.5%) and 97.1% (93.1–101.0%) in Gaya, Tessaoua and Agadez, respectively. No adverse events were observed. Ten mutations (SNP) were found, including 7 synonyms (K248K, G690G, E691E, E612E, C469C, G496G, P718P) and 3 non-synonyms (N594K, R255K, V714S). Two mutations emerged: N594K and V714S. The R255K mutation detected in Southeast Asia was also detected. The *pfdhpsK540E and pfdhfrI164L* mutations associated with high levels of resistance are absent. There is a reversal of chloroquine resistance.

**Conclusion:**

The study findings indicate that AL is effective and well tolerated for the treatment of uncomplicated malaria in three sites in Niger. The emergence of a *pfk13* mutation requires additional testing such as the Ring Stage Assay and CRISPR/Cas9 to confirm the role of these emerging mutations.

*Trial registration* NCT05070520, October 7, 2021.

**Supplementary Information:**

The online version contains supplementary material available at 10.1186/s12936-024-04945-8.

## Background

Malaria is a major public health issue in Niger. In 2020, the Niger Malaria Control National Strategic Plan reported 5827 deaths attributable to malaria and 5,141,257 confirmed malaria cases, for an incidence of 19,200 cases per 100,000 inhabitants [1]. *Plasmodium falciparum* is the predominant species in Niger, representing 98.3% of cases versus 1.7% for *Plasmodium malariae* [2].

Malaria treatment is based on the principle of early diagnosis and rapid management with effective drugs, mostly artemisinin-based combination therapy (ACT) [3]. The national malaria strategic plan recommends the following artemisinin-based combinations for the management of uncomplicated malaria: artemether-lumefantrine (AL), artesunate–amodiaquine (ASAQ), dihydroartemisinin–piperaquine (DP) and artesunate–pyronaridine [4]. However, AL is the most commonly used combination in health structures in Niger.

The use of ACT is threatened by the emergence and spread of artemisinin resistance. The emergence of resistance to artemisinin in Southeast Asia in 2008 [5] has heightened concern for the spread of resistant malaria parasites to Africa, as has resistance to chloroquine and sulfadoxine-pyrimethamine (SP). Recent studies reported the emergence of artemisinin resistance in East [6]—and Central [7] Africa. The World Health Organization (WHO) recommends that malaria-endemic countries evaluate the efficacy of their anti-malarials for the treatment of uncomplicated malaria every 2 years to allow the early detection of resistance and provide evidence for guiding national malaria treatment policy [8].

Recent therapeutic efficacy studies in Niger reported PCR-corrected efficacies of over 94% for AL. In 2017, the PCR-corrected AL efficacy was 96.2% in Dogondoutchi and 100% in Birni N’Gaouré [9]. In another study, conducted in 2013 at Maradi, the efficacy of AL after PCR correction was 99% [10]. Finally, in 2011, the efficacy of AL during a study conducted in Gaya revealed an efficacy of 94.8% [11].

Characterization of molecular markers of resistance in samples collected in therapeutic efficacy studies facilitates interpretation of clinical data. In 2013, a study of the *pfk13* gene associated with artemisinin resistance revealed a large polymorphism of this marker in Niger [12]. Its analysis in asymptomatic populations had shown 13 mutations (M472I; Y558C; K563R; P570L; A578S; P615S; I465I; C469C; R471R; L488L; G496G; V510V and Y630Y) of which six were nonsynonymous mutations (M472I; Y558C; P570L; A578S; P615S; I465I). Eight of these mutations (M472I; Y558C; K563R; P570L; P615S; L488L; V510V and Y630Y) were specific to Niger, among which there were five nonsynonymous mutations (M472I; Y558C; K563R; P570L; P615S) [12]. However, an analysis of this gene in a therapeutic efficacy study comparing AL and ASAQ in 2011 in Gaya found five mutations (R528K, A569G, V637I, C469C and Q613Q) including three nonsynonymous (R528K, A569G and V637I) and two synonymous (C469C and Q613Q) [13]. A nonsynonymous mutation (*Pfk13A569G*) was selected by ASAQ*.* However, AL did not select any mutation [13].

In 2017, a study of *pfdhfr* and *pfdhps* markers associated with SP resistance showed 97.4% prevalence for *pfdhfrS108N*, 92.6% for *PfdhfrC59R* and 85.0% for *PfdhfrN51I* in [14]. A 2018 study showed a prevalence of 59.0% for *PfdhpsA437G* and 43.1% for *PfdhpsS436A* [15]. In 2008, there was a statistically significant increase in prevalence of *pfcrtK76T* and *pfmdrA86N* markers associated with chloroquine and amodiaquine resistance to 32.4% and 17.4%, respectively [16].

This study was thus designed to evaluate the therapeutic efficacy and tolerability of AL and measure the prevalence of molecular markers associated with reduced susceptibility and resistance to AL, thereby allowing the Niger National Malaria Control Program (NMCP) to review and/or adapt its malaria control strategy.

## Methods

### Study sites

**Fig. 1:**
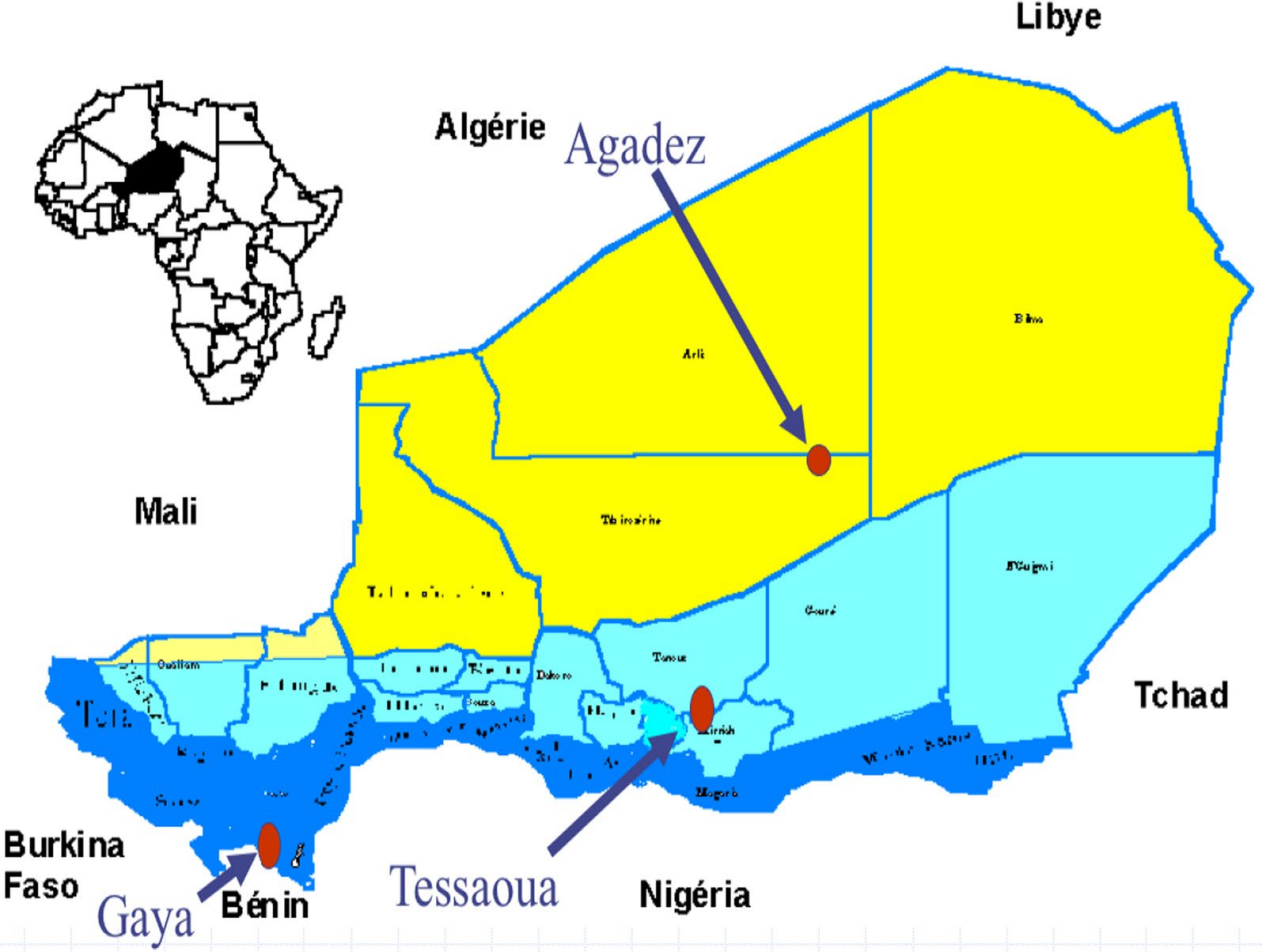
The three study sites (as red dots) used in the Niger therapeutic efficacy study, 2020 (Niger 2017–2022 National Malaria Strategic Plan)

This study was conducted in three sentinel sites in Niger: Centre de Santé Intégré (CSI) of Gaya in Dosso region, CSI of Tessaoua in Maradi region and CSI of Dagamanet in Agadez region, each located in different epidemiological facie of malaria in Niger. Malaria transmission is hypo-endemic in Dagamanet, meso-endemic in Tessaoua and hyper-endemic in Gaya (Fig. 1).

### Study design and participant recruitment

The efficacy of AL was assessed in a multi-center prospective one-arm study conducted from September 1 to October 31, 2020, as per the WHO 2009 28-day protocol for surveillance of anti-malarial drug efficacy [8]. In Niger, the treatment failure rate for AL is estimated at 5%. With a confidence level of 95% and an error margin of 5%, a sample size of 73 patients per site was calculated using the Schwarz formula and was increased by 20% to account for potential loss-to-follow-up and withdrawals during the 28-day follow-up period. The minimum sample size was thus set at 88 patients per site.

Participants were recruited among children aged 6 months to 15 years of age presenting at the sentinel site clinics with axillary temperature ≥ 37.5 °C or a history of fever in the past 24 h, P*. falciparum* parasitaemia between 1000–200,000 asexual parasites/μL, brachial circumference > 125 mm or a weight-to-height ratio (W/H Z-score) > − 2 standard deviations, and haemoglobin levels > 6 g/dL. Only patients with a capacity to take oral medications, whose parents/guardians are able and willing to adhere to the protocol for the duration of the study, and who signed the informed consent were enrolled. Malnourished children were not included, nor were patients with a history of anti-malarial treatment in the past 2 weeks, those with general clinical warning signs or signs of severe malaria, those with mixed infection or monospecific infection with another *Plasmodium* species, or those with a history of hypersensitivity to any of the drugs tested.

### Participant treatment and follow-up

The AL used in the study was provided by the National Laboratory of Public Health and Expertise [LANSPEX (Laboratoire National de Santé Publique et d’Expertise)] of Niger, approved by the WHO and manufactured by IDA laboratory (https://www.idafoundation.org/fr/services-dapprovisionnement). The AL tablets were presented in blister packs of 6 and 12 (lot numbers KU142 and KU45, respectively) and bore an expiration date of January 2022.

AL was administered to study participants orally at a dose of 4 mg artemether and 24 mg lumefantrine per kilogram of body weight for 3 days. The medication was taken under direct observation. The patient was then monitored for 30 min to observe any potential drug rejections or adverse events. If rejected, another dose was administered. Any patient who persistently vomited (two or more times) was not enrolled in the study and was treated with injectable artesunate. After the first day of AL treatment, each participant was followed up on days. During each visit, each child underwent a complete physical examination with axillary temperature measurement, a laboratory test to check parasitaemia with thick and thin blood smears and a dried blood sample was collected on filter paper for DNA. All blood specimens were collected by finger prick. In the case of treatment failure, injectable artesunate or injectable artemether was administered to the child according to the Niger national malaria treatment guidelines [4]. The patient was then classified as a failure.

### Laboratory testing

Rapid patient screening was achieved through malaria rapid diagnostic testing. Thick and thin blood smears were prepared using capillary blood from each patient and thin blood smears were fixed with methanol. The slides were stained with 10% Giemsa and read under a microscope (magnification 100X with immersion oil). All slides were read by two microscopists. In case of a difference in parasite count of at least 20% between the two readings, a third reading was performed by another microscopist. Parasite density was calculated based on 8000 leukocytes/μL of blood. Capillary blood was also collected on Whatman 903 filter papers systematically in all patients on the day of enrolment (day 0) and on follow-up days. Once dried, this blood was used for the genotyping of parasitic strains.

### Classification of patients after treatment

At the end of the 28-day follow-up period, treatment responses were classified according to the WHO definitions of clinical and parasitological criteria as either early treatment failure (ETF), late parasitological failure (LPF), late clinical failure (LCF), or adequate clinical and parasitological response (ACPR). The endpoints and the classification of responses to treatment are described in the WHO 2003 protocol [8].

### PCR correction

To distinguish recrudescent infections from reinfections, genotyping was performed with *msp1*, *msp2 and glurp* markers. The K1, MAD20 and RO33 loci of *msp1*, FC27, 3D7 loci of *msp2* were amplified and analysed (19). Parasite DNA extraction was performed using the QIAGEN Kit (QIAamp® DNA Micro-Kit. Reference Cat No. 56304). The extraction protocol, as described in the WHO bulletin [17] consisted of two major phases: (1) cell lysis and (2) DNA purification. This last phase included the following steps: enzymatic digestion with proteinase K, double washing with AW1 and AW2 buffers to remove proteins, contaminants, enzymes and other PCR inhibitors, and finally an elution step with AL buffer. The extracted DNA was finally stored at − 20 °C before PCR. Genetic polymorphism of the strains was analysed by amplifying the *msp1* block2 and the central variable region of *msp2* [18]. The specific primers of the different allele families (K1 and MAD20 for *msp1*, 3D7 and Fc27 for *msp2* and *glurp)* have made it possible to distinguish any reinfection from recrudescence [18]. Additional file 1 shows PCR corrections with the *msp1*, *msp2* and glurp markers at the three sites.

### Molecular markers of antimalarial drug resistance

The PCR/Sequencing method for *pfk13, pfdhfr, pfdhps, pfcrt* and *pfmdr* genes was used to study molecular resistance to anti-malarials [14, 19].

### Sequencing

The PCR2 products were then purified through enzymatic PCR cleanup using: 2 units of Exonuclease 1 (USB Corporation, Cleveland, OH), 1 unit of Shrimp Alkaline Phosphatase (USB Corporation) and 1.8 μL of double distilled H_2_O to which 8μL of the PCR2 product were added. This mixture was incubated at 37 °C for 15 min followed by 15 min at 80 °C to inactivate all enzymes. Each PCR product was sequenced using the two PCR primers to generate the sense-antisense (forward and reverse) sequences, using the Sanger method (CNR Malaria France).

### Statistical analysis

Clinical data were collected on paper and then double entered onto a tablet with the KoboCollect software to reduce the chance of error. Data was analysed using the EPI INFO 7.0.

Both per protocol and Kaplan–Meier analyses were conducted to evaluate the study outcome data. The main difference between the two is the Kaplan–Meier approach takes into account patients who were excluded from the study due to reinfection and censors them at the day of reinfection, whereas the per protocol approach removes those with reinfection from the analysis altogether in the PCR-corrected calculations.

Classification of responses to treatment was done using the WHO standard definitions. Prevalences were compared using the chi-square test. The ANOVA, Mann–Whitney U, or Kruskal–Wallis tests compared the averages with a 5% margin of error. The Kaplan–Meier curve was used to analyse parasite clearance.

### Ethical considerations

This study has received approval from the Ministry of Public Health Ethics Advisory Committee for Health by letter with reference No. 033/2020/CNERS. The Population Services International Research Ethics Committee determined that the activity was public health surveillance. Informed consent was obtained from the patient’s parent or legal guardian. The consent forms were dated and signed by both parties. The data collected was kept anonymous and confidential.

## Results

### Baseline characteristics of study participants

A total of 318 patients were screened for the study: 110 in Agadez; 113 in Tessaoua; and 95 in Gaya. Of these, 255 patients were enrolled: 80 in Agadez; 85 in Tessaoua; and 90 in Gaya. Average patient age was 8.9 years and the sex ratio (M/F) was 1.13. All enrolled patients had a monospecific *P. falciparum* infection. The average haemoglobin was 9.28 g/dL [95% CI 6.92–9.36]. The gametocyte index was 0.03 (8/255), specifically 0.01 (1/80) in Agadez, 0.03 (3/90) in Gaya, and 0.04 (4/85) in Tessaoua. Table 1 summarizes the main patient characteristics at day 0 at each of the three sites.

**Table 1:** Study patient characteristics at Day 0

Characteristics	Agadez	Tessaoua	Gaya	Total
Patients examined	110	113	95	318
Patients enrolled	80	85	90	255
Reached study endpoint	70	83	90	243
Average age (years)	8	10	9	8.9
Average weight (kg)	25.26	23.35	23.88	24.13
Average temperature (°C)	38.8	38.4	38	38.4
Sex ratio (M:F)	1.66	1.04	0.88	1.13
Parasite density (P/μL)	46,807.75	13,700.00	52,330.66	
Average gametocytemia	0.01	0.04	0.03	0.03
Average haemoglobin: (g/dL)	9.57	9.06	8.55	9.28

### Efficacy of artemether lumefantrine

**Fig. 2:**
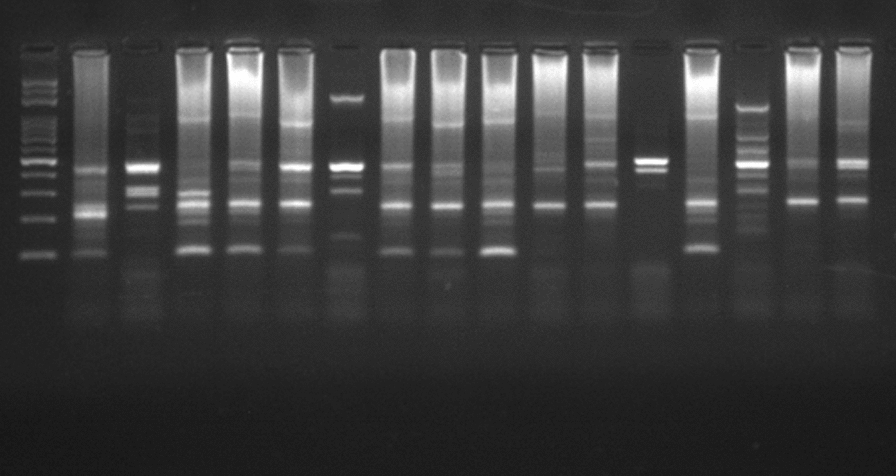
Genetic polymorphism of strains with the *glurp* marker

**Fig. 3:**
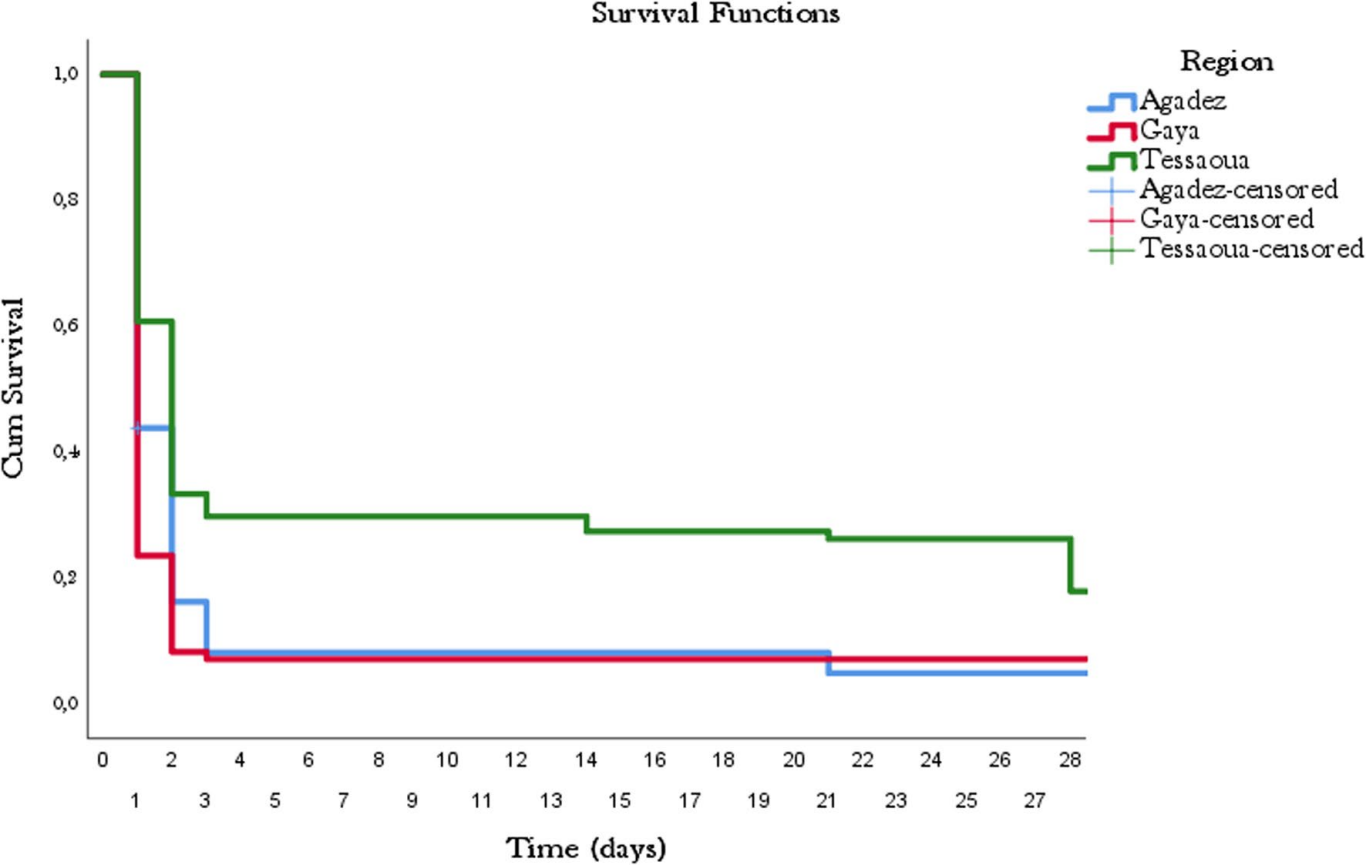
Kaplan–Meier Curve for all three study sites

The adequate clinical and parasitological response after correction was 97.05%, 92.18%, 98.87% in Agadez, Tessaoua and Gaya, respectively. Figure 2 shows genetic polymorphism of strains with the *glurp* marker and Table 2 shows treatment responses before and after PCR correction at each site. The Kaplan–Meier curve illustrates the survival of parasites after AL administration. Figure 3 shows the fraction of live parasites in patients during 28 days of follow-up in Agadez; Téssaoua and Gaya (Fig. 3). No treatment-related adverse events were reported during the study at any of the three sites, indicating that AL was well tolerated by patients.

**Table 2:** Uncorrected and PCR-corrected treatment outcomes at day 28 for AL, Niger 2020

Outcomes	Agadez	Tessaoua	Gaya
Number of patients	70	83	90
Number of recrudescence	2	5	1
Number of re-infections	2	19	1
Number of ACPRs	66	59	88
% Early treatment failure	0	0	0
% Late treatment failure	5.71	19.28	1.12
% Response before PCR correction	94.28 [88.8–99.7]	71.08 [60.5–81.7]	97.78 [94.3–101.2]
% Response after PCR correction	97.05 [93.1–101.0]	92.18 [85.0–98.5]	98.87 [96.4–101.0]

### Molecular markers of drug resistance

The main genes that were sequenced using the Sanger method were: *pfk13*, *pfdhfr, pfdhps, pfcrt*, and *pfmdr*. Two hundred seventy-four samples of which 250 from day 0 and 24 from the day of failure were sequenced.

### Molecular resistance to artemisinin

The Kelch gene analysis revealed 10-point mutations (SNPs) including three nonsynonymous mutations: N594K, R255K, V714S and seven synonymous mutations: K248K, G690G, E691E, E612E, C469C, G496G, P718P. The prevalence of the R255K mutation was 1.61% (4/248) and that of the C469C mutation was 0.81% (2/248). That of all the other mutations was 0.40% (1/248). The prevalence of the wild allele was 95.96%. The N594K and V714S nonsynonymous mutations have never been described elsewhere. These are new mutations that first emerged in Niger. On the other hand, the R255K nonsynonymous mutation observed in Southeast Asia first emerged in Niger. Nine-point mutations were observed before treatment, including three nonsynonymous and six synonymous mutations. These are K248K, G690G, E691E, C469C, G496G, P718P, N594K, R255K and V714S. A single synonymous mutation was observed after treatment, the E612E mutation. Therefore, artemisinin treatment did not select nonsynonymous mutations in the kelch gene.

### Molecular resistance to sulfadoxine and pyrimethamine

The prevalence of *pfdhfr* point mutations associated with pyrimethamine resistance were 88.85% (239/269) for *pfdhfrN51I*, 92.44% (30/269) for *pfdhfrC59R* and 94.44% (255/269) for *pfdhfrS108N*. The *pfdhfrI164L* mutation associated with a high level of pyrimethamine resistance was absent in our samples. The prevalence of the triple *pfdhfr* mutation was 85.24% (231/269). The prevalences of point mutations in the *pfdhps* gene, associated with sulfonamide resistance, were 50.00% (68/136) for *pfdhpsS436A*, 88.19% (127/144) for *pfdhpsA437G*, 15.79% (24/152) for *pfdhpsA613T,* 9.35% (13/139) for *pfdhpsI431V* and 8.86% (14/158) for *pfdhpsA581G.* The *pfdhpsK540E* mutation, associated with a high level of sulfonamide resistance, was also absent in our samples. The prevalence of dual mutations *pfdhpsS436A*/*pfdhpsA437G, pfdhpsA437G/pfdhpsA581G, pfdhpsA437G/pfdhpsA613T* and *pfdhpsA581G/pfdhpsA613T* were 90.77% (59/65), 3.77% (4/105), 2.83% (3/104) and 2.99% (8/268), respectively. The prevalence of the quadruple mutation *pfdhfrN51I*/*pfdhfrC59R/pfdhfrS108N/pfdhpsS436A* was 38.81% (104/268). That of the quadruple mutation *pfdhfrN51I*/ *pfdhfrC59R/pfdhfrS108N/ pfdhpsA437G* was 88.89% (104/117). The prevalence of the quintuple mutation *pfdhfrN51IpfdhfrC59R/pfdhfrS108N/pfdhpsS436A*/*pfdhpsA437G* was 93.88% (46/49).

### Molecular resistance to amino quinoline

The prevalence of the *pfcrtK76T* mutation associated with chloroquine resistance was 14.17% (35/247). That of the *pfmdrN86Y* and *pfmdrY184F* mutations associated with amodiaquine resistance were 5.34% (14/262) and 60.03% (159/262), respectively. The double mutations *pfmdrN86Y/pfmdrY184F* and *pfmdrN84F*/*pfmdrY184F* have a prevalence of 5.34% (14/262) and 0.72% (2/276), respectively. Table 3 shows the distribution of the prevalence of all resistance markers by site.

**Table 3:** Prevalence of resistance markers by site

Selection of molecular resistance markers	Agadez (%)	Tessaoua (%)	Gaya (%)
*pfK13*			
*pfK13N594K* (%)	0 (0/75)	0 (0/88)	1.18 (1/85)
*pfK13R255K* (%)	2.67 (2/75)	0 (0/88)	2.35 (2/85))
*pfK13V714S* (%)	1.33 (1/75)	0 (0/88)	0 (0/85)
*pfK13K248K* (%)	0 (0/75)	1.14 (1/88)	0 (0/85)
*pfK13G690G* (%)	0 (0/75)	1.14 (1/88)	0 (0/85)
*pfK13E691E* (%)	0 (0/75)	1.14 (1/88)	0 (0/85)
*pfK13C469C* (%)	0 (0/75)	0 (0/88)	1.18 (1/85)
*pfK13G496G* (%)	0 (0/75)	0 (0/88)	2.35(2/85)
*pfK13P718P* (%)	0 (0/75)	0 (0/88)	1.18 (1/85)
*pfK13E612E* (%)	0 (0/75)	1.14 (1/88)	0 (0/85)
*Wild pfK13* (%)	96 (72/75)	95.45 (84/88)	91.76 (78/85)
*Pfcrt*			
*pfcrtK76T* (%)	13.51(10/74)	10 (9/90)	19.28(16/83)
*Pfmdr*			
*PfmdrN86Y* (%)	5.48 (4/73)	1.02 (1/98)	9.89 (9/91)
*pfmdrY184F* (%)	58.90 (43/73)	50 (49/98)	73.63 (67/91)
*pfmdrS1034FC* (%)	0 (0/73)	0 (0/98)	0 (0/91)
*pfmdrD1246Y* (%)	0 (0/73)	0 (0/98)	0 (0/91)
*Pfdhfr*			
*PfdhfrN51I* (%)	93.51 (72/77)	86 (86/100)	88.04 (81/92)
*PfdhfrC59R* (%)	94.81 (73/77)	90 (90/100)	94.57 (87/92)
*PfdhfrS108N* (%)	97.40 (75/77)	90 (90/100)	96.74 (89/92)
*PfdhfrI164L* (%)	0 (0/77)	0 (0/100)	0(0/92)
*Pfdhps*			
*pfdhpsI431V* (%)	4.26 (2/47)	16.98 (9/53)	5.13 (2/39)
*PfdhpsS436A* (%)	43.18 (19/47)	65.38(34/52)	37.5 (15/40)
*PfdhpsA437G* (%)	94.12 (48/51)	81.13 (43/53)	90 (36/40)
*pfdhpsA613T* (%)	31.11 (14/45)	4.55 (3/66)	17.07 (7/41)
*pfdhpsA581G* (%)	10.64 (5/47)	10 (7/70)	4.88 (2/31)
*PfdhpsK540E* (%)	0 (0/48)	0 (0/68)	0 (0/40)
Multiple mutations			
*pfdhfrN51I/pfdhfrC59R/pfdhfrS108N/pfdhpsS436A* (%)	100 (18/18)	92.31(24/26)	92.31 (12/13)
*pfdhfrN51I /pfdhfrC59R/pfdhfrS108N/ pfdhpsA437G* (%)	95.56 (43/45)	78.38 (29/37)	91.43(32/35)
*pfdhfrN51I/pfdhfrC59R pfdhfrS108N/pfdhpsS436A/pfdhpsA437G* (%)	100 (17/17)	90 (18/20)	91.67 (11/12)

### Selection of mutations associated with antimalarial resistance

To analyse the selection of antimalarial resistant strains, the prevalence of markers before and after treatment was analysed. Neither the Kelch gene, nor the quadruple or fifth mutations were selected by treatment. Table 4 summarizes the selection of resistance markers by treatment.

**Table 4:** Selection of resistance markers

Selection of molecular resistance markers	Before treatment (%)	Post-treatment (%)
*pfK13*		
*pfK13N594K*	0.4 (1/230)	0 (0/18)
*pfK13R255K*	1.74 (4/230)	0 (0/18)
*pfK13V714S*	0.43 (1/230)	0 (0/18)
*pfK13K248K*	0.43 (1/230)	0 (0/18)
*pfK13G690G*	0.43 (1/230)	0 (0/18)
*pfK13E691E*	0.43 (1/230)	0 (0/18)
*pfK13C469C*	0.43 (1/230)	0 (0/18)
*pfK13G496G*	0.87 (2/230)	0 (0/18)
*pfK13P718P*	0.43 (1/230)	0 (0/18)
*pfK13E612E*	0 (0/230)	5.56 (1/18)
*Wild* *pfK13*	94.35 (217/230)	94.44 (17/18)
*Pfcrt*		
*pfcrtK76T*	14.54 (33/277)	10 (2/20)
*Pfmdr*		
*PfmdrN86Y*	94.21 (228/242)	0 (0/20)
*pfmdrY184F*	61.57 (149/242)	50 (10/20)
*pfmdrS1034FC*	0 (0/222)	0 (0/20)
*pfmdrD1246Y*	0 (0/224)	0 (0/20)
*Pfdhfr*		
*PfdhfrN51I*	89.92 (223/248)	76.19 (16/21)
*PfdhfrC59R*	93.15 (231/248)	86.36 (19/22)
*PfdhfrS108N*	95.16 (236/248)	86.36 (19/22)
*PfdhfrI164L*	0 (0/248)	0 (0/22)
*Pfdhps*		
*pfdhpsI431V*	9.85 (13/132)	0 (0/7)
*PfdhpsS436A*	48.82 (62/127)	66.67 (6/9)
*PfdhpsA437G*	87.50 (119/136)	100 (8/8)
*pfdhpsA613T*	13.99 (20/143)	44.44 (4/9)
pfdhpsA581G	8.72 (13/149)	11.11 (1/9)
PfdhpsK540E	0 (0/148)	0 (0/9)
Multiple mutations		
*pfdhfrN51I/pfdhfrC59R/pfdhfrS108N/pfdhpsS436A*	96.15 (50/52)	80 (4/5)
*pfdhfrN51I/pfdhfrC59R/pfdhfrS108N/ pfdhpsA437G*	90.83 (99/109)	62.50 (5/8)
*pfdhfrN51I/pfdhfrC59R/pfdhfrS108N/pfdhpsS436A/pfdhpsA437G*	95.56 (43/45)	70 (3/4)

## Discussion

This single-arm prospective study evaluated the therapeutic efficacy and tolerability of AL at the Agadez, Tessaoua, and Gaya sentinel sites in Niger. Mutations in the *pfk13*, *pfdhfr, pfdhps, pfcrt*, and *pfmdr* genes were also analysed to search for molecular markers of resistance to artemisinin, pyrimethamine, sulfonamides, chloroquine, and amodiaquine.

The WHO recommends the reported efficacy for AL at day 28 be lower than 90% to warrant considering a change in first-line treatment. This study shows that AL treatment for the management of uncomplicated *P. falciparum* malaria cases is effective in Niger and can continue to be used. All three study sites demonstrated an AL efficacy of over the WHO threshold, with ACPRs in Agadez, Tessaoua, and Gaya of 97.05%, 98.87% and 92.18%, respectively. Several studies have evaluated the efficacy of AL in Niger and the sub region and have demonstrated its continued efficacy in West Africa: 91% efficacy in Sélingué, Mali in 2015 [20], 96.7% and 96.3% efficacy in Bohicon and Kandi, Benin, respectively, in 2018 [21], 98.8% efficacy in Kédougou, Senegal, in 2018 [22] and 98.8% efficacy in Abidjan, Côte d’Ivoire, in 2016 [23]. In addition to its efficacy, this molecule is well tolerated by patients and no adverse events were reported during the study.

This analysis of molecular resistance markers reveals several highlights including the emergence of two novel non-synonymous mutations in the *pfk13* gene: N594K and V714S. The *pfk13* gene polymorphism has been studied in Niger [12], in Africa [13] and globally but these two mutations have never been reported in any setting [19]. The second highlight is the emergence of a Southeast Asian mutation in Niger: the R255K nonsynonymous mutation [19]. The third highlight is the increased prevalence of *pfk13* gene mutations. A previous study on polymorphisms of this gene revealed very low prevalence, rarely exceeding 0.1%. This study shows mutations with higher prevalence like that of R255K, which is 1.61%. Further research is warranted to determine whether this is the impact of the selection pressure exerted by ACT or the spread of artemisinin resistance in Africa. Indeed, Rwanda has already reported artemisinin-resistant strains of *P. falciparum* [7].

Regarding the *pfdhr* and *pfdhps* gene mutation analysis associated with resistance to 2,4-diaminopyrimidines and sulfonamides, respectively, the absence of *pfdhfrI164L* and *pfdhosK540E* mutations are observed to be associated with high levels of resistance to pyrimethamine and sulfonamides, respectively [15]. Thus, despite the high prevalence of *pfdhfr* and *pfdhps* gene mutations, the quintuple mutation is not as highly selected. The SP and sulfadoxine pyrimethamine plus amodiaquine (SPAQ) combinations are used for intermittent preventive treatment in pregnant women [24] and seasonal malaria chemoprevention in children aged 3 to 59 months [25], respectively. This study shows that these two malaria control strategies in pregnant women and children under 5 months of age are not yet threatened by anti-malarial resistance.

The analysis of *pfcrt* and *pfmdr* gene mutations associated with chloroquine and amodiaquine resistance, respectively, shows reversion of chloroquine resistance in Niger. The use of chloroquine was discontinued in 2005 due to a therapeutic failure rate of 40% and a high rate of the *pfcrtK76T* mutation [16]. A decade later, there was a significant decrease in the prevalence of the *pfcrtK76T* mutation from 32.4% in 2005 to 14.17% in 2020 [16]. The prevalence of the *pfcrt* mutation is not zero (14.17%) because chloroquine is still used in medicine to treat certain infections such as intestinal amoebiasis. Prevalences of *pfmdr* mutations also remain relatively low and warrant the continued use of SPAQ in seasonal malaria chemoprevention.

The key limitation inherent in this study is related to the administration of the second dose of AL, which was entrusted to caregivers without direct observation by healthcare workers. While this approach was practical, it introduced a potential vulnerability as it relied on caregivers’ responsibility. To address this limitation, the study team and healthcare workers implemented proactive measures to mitigate the possibility of misuse or non-administration. The study team and healthcare workers conducted interpersonal communication sessions with caregivers during consultations, emphasizing the critical importance of adhering to the prescribed timing and dosage for AL administration. Furthermore, they highlighted the potential impact of proper dosage adherence on the successful outcome of the treatment.

This study offers the advantage of providing regular updates to the NMCP on the efficacy and tolerability of ACT used for the management of uncomplicated malaria. For future studies, it will be crucial to assess the efficacy of other combinations of the national malaria case management policy in Niger.

## Conclusion

This study confirms that AL has an adequate clinical and parasitological response exceeding the 90% WHO-recommended cut off. The study also reveals the emergence of two new mutations and one Southeast Asian mutation in Niger. Finally, there is an absence of mutations associated with a high level of resistance to pyrimethamine and amodiaquine and a reversion of chloroquine resistance. The findings indicate that AL remains effective in the treatment of uncomplicated *P. falciparum* malaria in Niger and its continued use as first-line treatment is justified.

### Supplementary Information

**Additional file 1. **Results of recrudescence versus reinfection test (PCR msp1, msp2 and glurp) of failed samples. It shows PCR corrections with the msp1, msp2 and glurp markers at the three sites.

## Data Availability

The datasets will be available on the WWARN (https://www.wwarn.org).

## Abbreviations

ACT: Artemisinin-based combination therapy
NMCP: National Malaria Control Program
AL: Artemether–lumefantrine
WHO: World Health Organization
ASAQ: Artesunate–amodiaquine
DP: Dihydroartemisinin–piperaquine
SP: Sulfadoxine–pyrimethamine
CSI: Centre de Santé Intégré (Integrated Health Centre)
LANSPEX: Laboratoire National de Santé Publique et d’Expertise
ETF: Early treatment failure
LPF: Late parasitological failure
LCF: Late clinical failure
ACPR: Adequate clinical and parasitological response
